# Plan Adopted by the Institution for the Cure and Prevention of Contagious Fever in the Metropolis
*As this subject has lately attracted a suitable degree of notice, both in London and in the country, the Editors conclude, that the details of the plan which has actually been adopted in the metropolis, cannot fail to be acceptable to their Readers.


**Published:** 1802-12-01

**Authors:** 


### *Plan adopted by the Institution for the Cure and Prevention of Contagious Fever in the Metropolis.**

1. ALL Subfcribers of one guinea a year, or upwards; or of ten guineas or more, in one donation, fhall be Governors of this Inftitution.

2. The Inftitution fhall be under the direction of a Committee of 32, confifting of the Prefident, fix Vice-Prefidents, the Treafurer, and 24 other members, who fhall be elected by the Governors.

3. All poor perfons, labouring under infectious fever, and refiding within limits hereafter to be affigned, fhall be confidered as proper objects of this Charity.

4. Houfes of Recovery fhall be provided for the reception of thofe whom it may be thought necefsary to remove from their own habitations. They fhall be in airy fituations; fufficiently detached from other buildings; and in the neighbourhood of a populous diftrict of the town.

5. As far as may be practicable, the houfes fhall be divided into feparate apartments, to be appropriated to patients in the different ftages of fever.

6. Upon the recommendation of any one for relief by this Charity, notice fhall be immediately given to the Phyfician, and the patient may be admitted into the Houfe by an order for that purpofe, figned by the Phyfician.

7. A chair, provided with a moveable lining, or fome other means of conveyance, fhall be kept at the Houfe, in which all perfons ordered by the Phyfician to be removed fhall be carried thither at the expence of the Inftitution.

8. Regulations for the internal management of the Houfe fhall be prepared under the direction of the Committee, with the affiftance of the medical officers of the Inftitution.

9. When

---

* As this fubject has lately attracted a fuitable degree of notice, both in London and in the country, the Editors conclude, that the details of the plan which has actually been adopted in the metropolis, cannot fail to be acceptable to their Readers.

9. When the Phyſician ſhall think the removal of a fever patient unneceſſary, or when the fever ſhall have ceaſed in a dwelling-houſe, meaſures ſhall be adopted for the purpoſe of checking the progreſs of contagion, or preventing the renewal of its effects. The apartments ſhall be cleanſed and white-waſhed, and infected bed-clothes and apparel ſhall be purified or deſtroyed.

10. A ſtock of bed-clothes and apparel ſhall be provided, to conſiſt of ſuch articles as the Committee may direct, from which the objects of this charity ſhall be ſupplied when it may be neceſſary.

11. A general meeting of the Subſcribers ſhall take place twice every year, viz. on the firſt Friday in May, and on the firſt Friday in November. Special meetings of the Subſcribers ſhall be called by the Preſident, at the requeſt of the Committee, or of any ſeven Governors, ſeven days previous notice being given thereof, and of the buſineſs to be tranſacted. At the general meeting in May, the Preſident, Vice-Preſidents, Treaſurer, and other Members of the Committee, ſhall be anually elected; eight of the thirty-two Members of the Committee being to go out and to be replaced by eight other Governors of the Inſtitution.

12. The Committee ſhall meet on the laſt Friday of every month, and at leaſt three Members ſhall be neceſsary to conſtitute a meeting.

13. The Committee ſhall appoint all the officers and ſervants of the Charity. They ſhall form temporary regulations for the management of the Houſe, which ſhall be in force until the ſucceeding general meeting, but no longer, unleſs then confirmed.

14. The Committee ſhall from time to time publiſh a report of the ſtate of the Inſtitution.

15. The Treaſurer ſhall receive all ſums of money paid for the uſe of the Inſtitution, and ſhall give ſuch ſecurity for the faithful diſcharge of his office as the Committee ſhall think ſufficient. He ſhall make all payments ſanctioned by the Committee, and ſhall lay before them at each meeting a ſtatement of his accounts, and the ſame ſhall be audited and balanced, and ſubmitted to the General Meeting in May.

16. The Treaſurer ſhall appoint a Clerk for the purpoſe of collecting ſubſcriptions, and ſhall be reſponſible for his conduct. The clerk ſhall receive ſuch remuneration as the Committee ſhall think proper.

17. The Committee ſhall at each monthly or other meeting, appoint Directors of each Houſe, who ſhall continue in office until the next meeting of the Committee.

18. The Directors ſhall give orders for the purifying of clothes and apartments where the Phyſician reports it to be neceſſary; and when application is made for a ſupply of clothes, they ſhall give an order in writing for ſuch articles as they may deem requiſite.

19. They ſhall order a reward to ſuch amount (ſubject to the regulation of the Committee) as they may think proper, to be given after the ceſſation of fever, on condition that the rules preſcribed for cleanlineſs, ventilation, and the prevention of infection, have been faithfully obſerved. The reward ſhall be proportioned to the degree of previous danger, and the ſucceſs of the meaſures by which it ſhall have been counteracted.

20. The Directors, before every meeting of the Committee, ſhall cauſe the bed-clothes and apparel belonging to the Inſtitution to be examined, and ſhall report thereon to the Committee.

21. The Directors ſhall be authoriſed in all reſpects to aid the execution, and enforce the obſervance of the regulations of the Inſtitution; and they ſhall notice, and, if neceſſary, report to the Committee any irregularity or miſconduct on the part of the ſervants or patients of the Charity.

22. The attending Phyſician ſhall, upon receiving an application in behalf of any object of this Charity, aſcertain the ſtate of the ſick perſon either by perſonal inſpection, or by obtaining a ſatisfactory ſtatement of the caſe from a Phyſician or an Apothecary. If it be neceſſary, either on account of the extreme poverty of the patient, or of the crowded ſtate of his habitation, that he be removed to the Houſe, the Phyſician ſhall give an order to that effect.

23. The Phyſicians ſhall viſit each Houſe at ſuch times as may be deemed neceſſary by the Committee; and ſhall attend at their own houſes thoſe patients whom they may not think it proper to remove.

24. The Phyſicians ſhall keep accurate regiſters of the caſes of all in-patients admitted under their care, and of the remedies employed.

25. They ſhall alſo report the meaſures neceſſary to be adopted in places where the contagion ſubſiſts, or has appeared.

26. The Committee ſhall allot the portion of duty to be undertaken by the Phyſician.

27. An Apothecary ſhall be appointed for each Houſe, and ſhall reſide near the Houſe, which he ſhall attend at leaſt once every day, and at ſuch other times as the Phyſician ſhall appoint, and on all caſes of emergency.

28. He ſhall prepare the medicines for the patients, and ſhall attend attend at a certain hour for the purpofe of delivering thofe ordered for the out-patients.

29. The Apothecary fhall receive fuch compenfation for his attendance as fhall be fixed by the Committee.

30. The Secretary fhall iffue fummonfes for, and attend all meetings of, the Committee and Governors. He fhall enter in proper books an account of their proceedings, and fhall do fuch other bufinefs as the Committee may direct.

31. He fhall be entrufted generally with the care of the clothes and other things belonging to the Inftitution.

32. He fhall be under the direction of the Committee and Directors, and fhall fuperintend the execution of the meafures enjoined by them for cleanfing and purifying clothes and apartments.

33. He fhall report to the Directors the Phyfician's opinion as to the articles of clothing required, and fhall deliver none out of his cuftody but in confequence of an order figned by the Directors.

34. He fhall deliver wine only to thofe who produce an order figned by the Phyfician, fpecifying the name of the patient for whom it is ordered, and the exact quantity required.

35. He fhall from time to time vifit the apartments of any perfon to whom it fhall have been found neceffary to entruft bed-clothes or apparel, and fhall afcertain whether they are applied to the intended purpofe; and in cafe of any mifufe of them, he fhall immediately report the fame to the Directors.

36. He fhall lay before the Committee at each meeting an account of the articles of clothing, &c. in his poffeffion, and a lift of thofe lent, or given, by order of the Directors, and of thofe returned fince the preceding meeting of the Committee.

37. Before entering upon his office, he fhall give fecurity for his good conduct to fuch amount as the Committee fhall determine.

38. The fervants of each Houfe fhall confift of a Matron, who fhall fuperintend the domeftic concerns, and of fo many ordinary nurfes as may be abfolutely neceffary, together with a porter, and fuch extra attendants as from time to time the Committee fhall think requifite.

39. The porter of each houfe fhall affift in carrying thofe whom the Phyfician fhall have ordered to be removed to the Houfe, and fhall be otherwife employed as the Committee and Directors fhall appoint.

LIST

### LIST OF THE GENERAL COMMITTEE.

PRESIDENT.

HIS GRACE THE DUKE OF SOMERSET.

VICE PRESIDENTS.

The Lord Bifhop of Durham.

Lord Sheffield.

Sir Walter Farquhar, Bart.

Wm. Wilberforce, Efq. M.P.

Langford Millington, Efq.

Thomas Bernard, Efq.

TREASURER.

Mr. Richard Phillips.

COMMITTEE.

Thomas Baring, Efq.
Henry Boafe, Efq.
Thomas Bonar, Efq.
Anthony Clarke, Efq.
Patrick Colquhoun, Efq.
Edward Forfter, jun. Efq.
William Garrow, Efq.
Maxwell Garthfhore, M. D.
John Hingefton, Efq.
Alexander Marcet, M. D.
John Miller, Efq.
[illegible]mes Allan Park, Efq.
John Pearfon, Efq.
Mr. William Phillips.
Wm. Morton Pitt, Efq. M. P.
Thomas Pitt, Efq.
James Redit, Efq.
Chriftopher Stanger, M. D.
Samuel Thornton, Efq. M. P.
William Waddington, Efq.
John Walker, Efq.
John Warburton, Efq.
Robert Willan, M. D.
John Yelloly, M. D.

PHYSICIANS EXTRAORDINARY.

Robert Willan, M. D. F. A. S. Bloomfbury Square.

Chriftopher Stanger, M. D. Grefham Profeffor, and Phyfician to the Foundling Hofpital, Lamb's Conduit Street.

PHYSICIAN. William Pitts Dimsdale, M. D. Greville Street, Holborn.

APOTHECARY. Mr. Jofeph Dymond, No. 146, Holborn Bars.

SECRETARY. Mr. Charles Murray, No. 19, Greville Street.

INSPECTOR. James Wifeman.

COLLECTOR. Mr. William Bond Copeland, No. 1, Devonfhire Street, Red Lion Square.

